# Supplementary figures and images for: Complement propagates visual system pathology following traumatic brain injury
Source: J Neuroinflammation. 2024 Apr 17;21:98. doi: 10.1186/s12974-024-03098-4 (PMC11022420; doi:10.1186/s12974-024-03098-4)

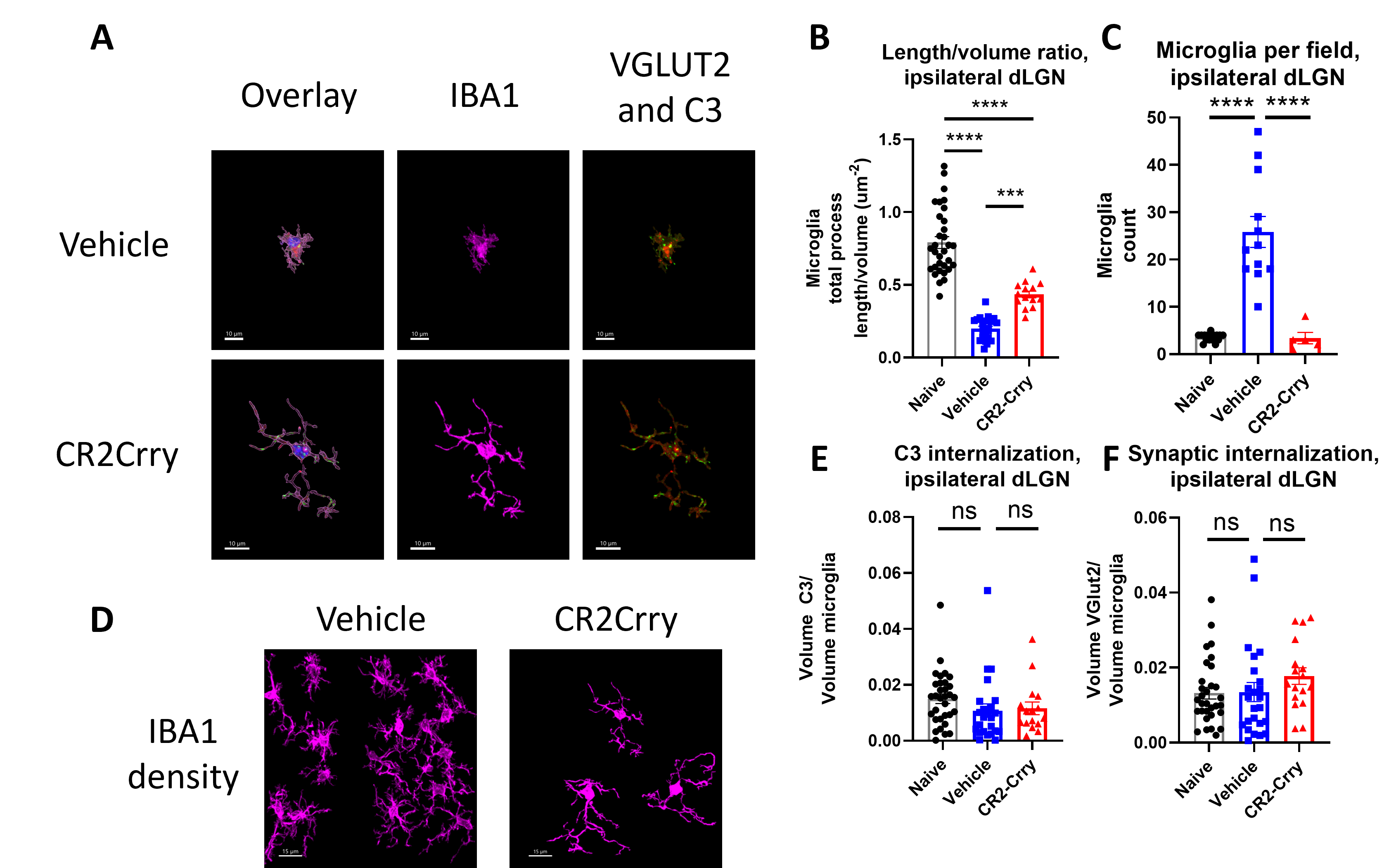

Supplement: Supplementary file 1 — Additional file 1. Inhibition of the complement system reduces microglia counts and partially attenuates morphological changes chronically in the dLGN ipsilateral to injury without affecting synapse or C3 internalization. A Representative microglial reconstructions with internalized VGLUT2 (green) and C3 (red), and microglial morphology (IBA1, magenta). Scale bar = 10 µm. B Microglia filament length to volume ratio in the ipsilateral dLGN. (C-D) Microglia count per 63 × high power field. Scale bar = 15 µm E Microglial internalization of VGLUT2 and (F) microglial internalization of C3 in the ipsilateral dLGN. B, C, E, F one-way ANOVA with Tukey correction for multiple comparisons. *p < 0.05, **p < 0.01, ***p < 0.001, ****p < 0.0001. Error bars = mean ± s.e.m. [file 12974_2024_3098_MOESM1_ESM.tif]
